# Intervention Packages for Early Visceral Leishmaniasis Case Detection and Sandfly Control in Bangladesh: A Comparative Analysis

**DOI:** 10.4269/ajtmh.18-0290

**Published:** 2018-11-19

**Authors:** M. Mamun Huda, Debashis Ghosh, Abdul Alim, Md. Almahmud, Piero L. Olliaro, Greg Matlashewski, Axel Kroeger, Dinesh Mondal

**Affiliations:** 1 Nutrition and Clinical Services Division, International Centre for Diarrhoeal Disease Research, Bangladesh (icddr,b), Dhaka, Bangladesh;; 2 Special Programme for Research and Training in Tropical Diseases (TDR), World Health Organization, Geneva, Switzerland;; 3 Department of Microbiology and Immunology, McGill University, Montreal, Canada;; 4 Centre for Medicine and Society, University Medical Centre Freiburg, Freiburg, Germany

## Abstract

We compared the efficacy of three intervention packages for active case detection (ACD) of visceral leishmaniasis (VL)/post–kala-azar dermal leishmaniasis (PKDL) combined with sandfly control around an index case. The packages were 1) no kala-azar transmission activity involving indoor residual spraying (IRS) with deltamethrin, peri-domestic deployment of larvicide with temephos, and house-to-house search for cases; 2) fever camp (FC) plus durable wall lining (DWL) with deltamethrin; and 3) FC plus insecticide (deltamethrin) impregnated bed-nets (ITN) around an index case. Fever camp includes 1-day campaign at the village level to screen and diagnose VL, PKDL, leprosy, malaria, and tuberculosis among residents with chronic fever or skin disease. Efficacy was measured through yield of new cases, vector density reduction, and mortality at 1, 3, 6, 9, and 12 months following intervention. Fever camp + DWL was the most efficacious intervention package with 0.5 case detected per intervention, 79% reduction in vector density (incidence rate ratio [IRR] = 0.21, *P* = 0.010), and 95.1% (95% confidence interval: 93.4%, 96.8%) sandfly mortality at 12 months. No kala-azar transmission activity was efficacious for vector control (74% vector reduction, IRR = 0.26, *P* < 0.0001 at 9 months; and 84% sandfly mortality at 3 months), but not for case detection (0 case per intervention). Fever camp + ITN was efficacious in detection of VL/PKDL cases (0.43 case per intervention), but its efficacy for vector control was inconsistent. We recommend index case–based FC for ACD combined with DWL or IRS plus larvicide for sandfly control during the consolidation and maintenance phases of the VL elimination program of the Indian subcontinent.

## Introduction

Visceral leishmaniasis (VL), also known as kala-azar, is a vector-borne disease transmitted by the female sandfly *Phlebotomus argentipes*. Chronic fever, enlargement of the spleen, darkening of the skin, anemia, and thrombocytopenia are the main clinical and laboratory features of VL. The disease is fatal if not treated in time. In 2006, the estimated burden of VL was about 50,000 new cases per year with a mortality rate of 1–20%. Although some 100 countries report VL,^1^ the most affected countries are Bangladesh, India, Nepal, Sudan, South Sudan, and Brazil.^2^ However, the situation has changed with the implementation of the VL elimination program in the Indian subcontinent. The elimination target is a VL incidence of less than one per 10,000 people at the upazila (subdistrict), district, and block levels, respectively, in Bangladesh, Nepal, and India. Nepal and Bangladesh have already achieved the target and India is very close to it.

In 2006, in Bangladesh the estimated annual incidence was 12,000–24,000 cases with a case fatality rate of 1%;^1^ about 130 upazilas of 45 districts reported VL cases at the beginning of the century.^2^ Fortunately, the VL burden in Bangladesh has declined as a result of the huge efforts of the National Kala-azar Elimination Program (NKEP)^3^ and the country achieved its target in 2016 (personal communication with Director, Communicable Disease Control, Directorate General of Health Services, Government of Bangladesh).

The first outbreak of VL was reported in 1824; since then, periodic peaks of VL have occurred every 10–12 years, demonstrating the cyclic nature of the epidemiology of VL in the Indian subcontinent.^4^ This also emphasizes the need for new VL control strategies after achieving the elimination target in each member countries of the subcontinent so that subsequent VL peaks could be prevented.

The elimination program has three phases: attack (which is successfully over in Bangladesh and Nepal), consolidation, and maintenance phases. Early diagnosis and proper treatment of VL cases involves house-to-house active case search, and sandfly control with integrated vector control management mainly with blanket (all households in a village). Indoor residual spraying (IRS) with insecticides has been a key element of the elimination program during the attack phase.^5^ Now, new strategies for the consolidation and maintenance phases should be identified and tested: first, because the former approach is no longer cost-effective now that the case load has decreased; second, because in the attack phase, identifying cases with post–kala-azar dermal leishmaniasis (PKDL) was not emphasized. A recent study in Bangladesh demonstrated that PKDL cases could transmit *Leishmania donovani* to the sandfly, lending support to the opinion that PKDL cases could be interepidemic reservoirs.^6^

**Figure 1.**
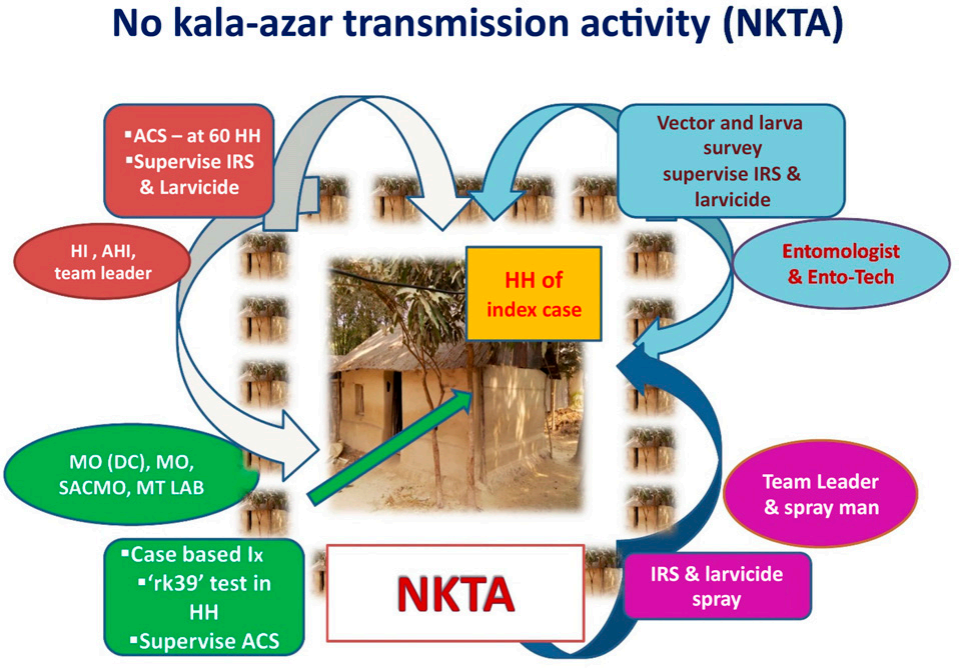
No kala-azar transmission activity in NKEP in Bangladesh. ACS = active case search; AHI = assistant health inspector; HH = household; HI = health inspector; IRS = indoor residual spraying; MO = medical officer; MO (DC) = MO (disease control); MT LAB = medical technologist (laboratory); NKEP = national kala-azar elimination program; NKTA = no kala-azar transmission activity; SACMO = sub-assistant community medical officer. (Source: National Guideline for Kala-azar Case Management in Bangladesh, 2016, NKEP, Communicable Disease Control, Directorate General of Health Services, Bangladesh.) This figure appears in color at www.ajtmh.org.

The current practice of the national program in Bangladesh is the recently introduced “no kala-azar transmission activity (NKTA).”^7^ The NKTA includes house-to-house search for VL and PKDL cases, IRS, and the use of larvicides in suspected vector breeding places in 60 houses around the house of a recently reported VL case (index case) (Figure 1).^7^ However, its yield and effectiveness for detecting VL cases and reducing sandfly density have not been properly quantified and compared with other alternatives. In terms of active case detection, fever camps (FCs) have been found to be an effective approach to identify not only VL and PKDL cases^8^ but also tuberculosis, leprosy, enteric fever, and malaria cases,^9^ and can be implemented by the program because its operation cost is low.^10^ In terms of vector control, impregnation of existing bed-net with a slow-release insecticide tablet (K-O Tab 1-2-3) (Bayer Environmental Science, Bayer [Ply] Ltd., reg. no. 1968/011192/07, 21 Isando, South Africa, CODE 05682036 C) is an effective tool for reducing sandfly density and VL burden when it is applied in all villages of a union,^11,12^ but its efficacy remains to be established when implemented in all houses within 100-m radius of the house of a VL index case. Durable wall lining (DWL) impregnated with insecticide (deltamethrin) has been found to be effective against *Anopheles* mosquitoes^13^ and sandflies.^14^ Durable wall lining could be a suitable tool for NKTA, replacing IRS in the future.

Thus, combined FC, including bed-net impregnation or DWL, could be an effective strategy for improved VL and PKDL case detection and sandfly control. In the present study, we investigated the efficacy of three different index case–based intervention packages for early detection of cases with VL and PKDL and sandfly control in support of the NKEP in Bangladesh.

## Methods

### Study area and duration.

The study was conducted in VL-endemic villages with a recent VL case in highly VL-endemic upazilas (subdistricts), Fulbaria and Trishal of Mymensingh district, from September 2015 to June 2017. The study activity period of 18 months included 4 months of pre-intervention activities, 2 months of intervention activities, and 12 months of follow-up activities.

### Study design.

**Figure 2.**
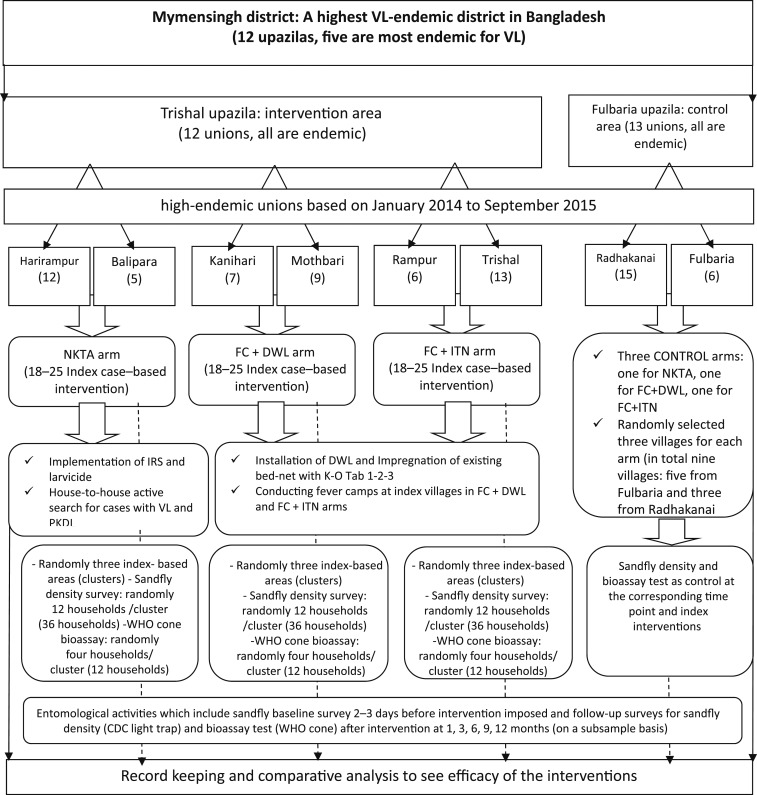
Study design. CDC = Centers for Disease Control and Prevention; FC + DWL = fever camp and installation of durable wall lining; FC + ITN = fever camp and insecticide-treated net; IRS = indoor residual spraying; NKTA = no kala-azar transmission activity; PKDL = post–kala-azar dermal leishmaniasis; VL = visceral leishmaniasis; WHO = World Health Organization.

This was a cluster-randomized controlled trial. A cluster represents the area (village/clusters) of an index case (a case of VL or PKDL) in study unions (lower administrative area of subdistricts in Bangladesh) of Trishal and Fulbaria (Figure 2). Trishal and Fulbaria were randomly assigned for intervention and control areas, respectively. Therefore, highly VL-endemic villages in Trishal were intervention areas and those in Fulbaria served as control areas.

*Selection of areas for index case–based intervention:* Highly VL-endemic unions from each upazila were identified based on the VL cases reported during January 2014 to September 2015. We calculated the VL incidence at the union level to stratify the study areas for index case–based intervention activities. In Trishal, we identified three pairs of unions with similar VL incidence and randomly assigned each of the three interventions to each pair. Similarly, a pair of unions with similar VL incidence was selected for control areas in Fulbaria (Figure 2). The villages of index cases in the selected arms were identified based on recently (September 2015 to February 2016) reported VL or PKDL cases for implementing intervention activities. Within the selected villages, 60 households around the index case (considered as an index case–based cluster) whose heads agreed to participate and signed the consent form were included in the study. We conducted FCs in all villages with index cases (Figure 2) in the intervention areas.

*Selection of households for entomological assessment:* We randomly selected three index case–based intervention clusters from each arm for entomological efficacy trials. Again, 12 households from each of the three clusters in each intervention arm were selected randomly, which yielded 36 households in each intervention arm. At the same time, three different matched control clusters, each having 36 households, were selected from control arms. In total, 72 (36 for intervention and 36 for control) households were enrolled in the study for sandfly density survey 2–3 days before starting the intervention and follow-up surveys at 1, 3, 6, 9, and 12 months. Similarly, 12 households for intervention and 12 households for control areas were selected for bioassay tests at 1, 3, 6, 9, and 12 months after the intervention (Figure 2). The sample size calculation is given later in the article.

### Operational definitions.

*Visceral leishmaniasis case:* Individuals exposed to a VL-endemic area, having fever for more than 2 weeks, and having an enlarged spleen were considered as suspected VL cases. A confirmed VL case was a suspected VL case with a positive rK39 test.

*Post*–*kala-azar dermal leishmaniasis case:* A PKDL suspect is a treated VL case with skin lesion with preserved skin sensitivity. A confirmed PKDL case is a PKDL suspect with a positive rK39 test.

*Leprosy case:* An individual with skin lesion with loss of skin sensitivity. A confirmed case of leprosy was a suspect with *Mycobacterium leprae* demonstrated in a skin specimen by microscopic examination.

*Tuberculosis case:* An individual with productive cough lasting more than 3 weeks. A confirmed case of TB was a TB suspect with *Mycobacterium tuberculosis* in the sputum by microscopy.

*Malaria case:* Fever or a history of fever within the last 48 hours plus a high index of suspicion based on time, place, and person (endemic zone, susceptible population, transmission season, etc.). A confirmed case of malaria was a malaria suspect with a positive rapid test for malaria.

*Index case:* A recently diagnosed case of VL or PKDL at the upazila health complex.

*Index household:* Household of the index cases was defined as the index household.

*Index-based areas/clusters:* Sixty households around the index case household were considered as an index-based cluster.

*Camp attendant:* The person who attended the camp with or without chronic fever (fever > 2 weeks) or skin lesion like PKDL.

*Camp participant:* The camp attendant who had chronic fever (fever > 2 weeks) or skin lesion like PKDL.

### Experimental interventions.

*No kala-azar transmission activity:* The program includes house-to-house active search for cases with VL and PKDL, implementation of IRS with deltamethrin, and deployment of larvicide (Temephos 50 EC, 5 mL/10 L) (Limbate 50 EC, D Limit Agro Product Limited, reg. no. 163, Bangladesh) at suspected sandfly breeding places in 60 households around the index household. This is based on the fact that a sandfly’s flight range is about 100 m. The number of households around an index case house is about 60 in Bangladesh (Figure 1).^7^

*Fever camp and installation of DWL:* This program includes a FC at the village of the index case for detecting VL/PKDL and other febrile and skin diseases (such as leprosy, tuberculosis, and malaria) plus installation of DWL impregnated with insecticides (deltamethrin, 170 mg a.i./m^2^) in 60 households around the IC’s household.

*Fever camp and insecticide-treated net (ITN):* This program includes a FC at the village of the index case for detecting VL/PKDL and other febrile and skin diseases (such as leprosy, tuberculosis, and malaria) plus impregnation of existing bed-nets with a slow-release insecticide tablet (K-O Tab 1-2-3 containing 0.4 g deltamethrin in a 1.6-g tablet) (ITN).

*Control:* Households in control clusters received IRS at the end of the study.

### Pre-intervention activities.

*Meeting and training:* We conducted this study in collaboration with the VL elimination program, Communicable Disease Control, Directorate General of Health Services, Bangladesh. Before starting the intervention, a meeting was organized with program managers at the subdistrict level and the central level to discuss the objective and methods of the study. In addition, the study staff received 1-day training on tracking index cases, interviews of study participants, and record keeping.

*Baseline sandfly density survey:* Before starting intervention activities, we conducted a sandfly density survey against which to quantify the efficacy of the vector control activities in each intervention arm. See the following paragraphs for a detailed analysis of the efficacy of vector control activities.

### Intervention activities.

Trained field research assistants were in contact with the two local hospitals (upazila health complexes) to track new VL/PKDL cases and to identify their home villages. The research team in collaboration with the program staff organized the corresponding intervention activities (see the following paragraphs) in these villages during January to February 2016. The team members for implementing the intervention activities included an entomologist, an entomological technician, a health assistant, IRS staff, a field research assistant, and a health worker together with the program staff.

*Implementation of NKTA:* After the identification of the index cases, the team visited their household for implementing the NKTA intervention (Figure 1),^7^ which included the following:

1. Applying IRS with deltamethrin in the living rooms and cattle shed of 60 households around the index household. This was conducted by trained spraying squads who also kept records
2. Identifying suspected vector breeding places in and around the 60 households and apply the larvicide temephos
3. Looking for suspected cases of VL and PKDL by using a structured questionnaire. Referring the suspected cases to the upazila health complex for further confirmation and management as appropriate

*Implementation of FC + DWL:* The research team (see previous paragraphs), in collaboration with well-trained community volunteers, implemented the intervention activities:

1. Installing the DWL in the main living room of the 60 households around the index household
2. Organizing a FC at the village of the index case (see the following paragraphs for details)
3. Keeping records

*Implementation of FC + ITN:* The research team implemented the following intervention activities in collaboration with the program staff:

1. Impregnation of the existing bed-nets of 60 households around the index household and recording the coverage using a structured questionnaire. New bed-nets were made available if a selected household had none
2. Organizing a FC at the index case village (see the following paragraph for details)
3. Keeping records

*Organization of index case–based fever camp:* We conducted a 1-day FC at the village of the index case to screen and diagnose VL, PKDL, leprosy, malaria, and tuberculosis among residents with chronic fever or skin disease. For conducing FC properly, we developed standard operating procedures for the FC and trained the camp team, which included one medical doctor, one laboratory technician, and one health assistant from the upazila health complex together with the research staff. The field research assistant and the health assistant visited the index case village to identify the camp place. The date of the camp was agreed upon with the program managers at the upazila health complex. Awareness activities (miking to invite to the FC) were carried out before the camp day with the support of local health functionaries and community volunteers. All camp attendants were recorded by the field research assistant during the camp; only subjects with fever lasting for more than 2 weeks or skin lesions suggestive of PKDL were enrolled as camp participants. The medical doctor did the physical examination of the camp participants and the medical technologist performed the rK39 test on participants with chronic fever and enlarged spleen. During the physical examination, the physician specifically looked for splenomegaly and PKDL-type skin lesions in subjects with a past history of VL. Newly detected cases of VL and PKDL were referred to the upazila health complex for further diagnosis, treatment, and clinical and biochemical monitoring investigations.

The rK39-negative febrile cases were screened for tuberculosis (sputum samples), malaria (rapid diagnostic test), typhoid, and other febrile illnesses. Suspected PKDL cases who tested negative on rK39 were investigated for leprosy. All the cases were referred to the upazila health complex for confirmatory diagnosis and treatment.

### Post-intervention activities.

The sandfly density measurements and the WHO cone bioassay test on the intervention surfaces were carried out at 1, 3, 6, 9, and 12 months after intervention.

### Entomological activities.

*Sandfly density survey:* Sandfly collection, preservation, and identification were conducted using the WHO/TDR monitoring and evaluation tool kit for IRS.^15^ Briefly, there were 36 households in each intervention arm, with 36 corresponding control households for sandfly density measurement. The density measurements were carried out in two consecutive nights for each household. Each night, trained personal installed a Centers for Disease Control and Prevention (CDC) light trap only in the main bedroom of each household from 6 pm to 6 am. Light traps were placed at the corner of the bedroom 2 cm away from the wall with the bottom of the sac 6 cm from the floor. Sandfly density was expressed as the number of female *P. argentipes* per household or trap per night. Sandfly identification was carried out as follows:

1. Sandflies were segregated and labeled by batch, indicating the date and the pre-printed batch numbers. Sandfly numbers and sex were identified under the microscope on mounted specimens.
2. Morphological identification was carried out in the field using the following criteria:
  - *Phlebotomus argentipes:* Black thorax + silver shining of the tarsal tip of the leg + 3 mm
  - *Phlebotomus papatasi:* Brown to yellow thorax + 3 mm
  - *Sergentomyia* spp.: 1–2 mm
3. Sex and physiological status
  - Males: external genitalia with claspers
  - Females: without claspers
    Physiological status: blood fed, unfed, gravid (no undigested blood)

Sandfly data were recorded by using structured record forms.

*Bioassay:* The cone bioassay tests were performed according to the WHOPES method with 12 randomly selected households out of 36 households for sandfly density measurement per intervention at 1, 3, 6, 9, and 12 months after the intervention. For IRS and DWL, the test covered four wall surfaces for IRS and DWL, but for ITN it covered five surfaces of a bed-net, including four sides and the top of the bed-net. The bioassay was carried out using the WHO/TDR monitoring and evaluation tool kit for IRS.^15^

### Sample size calculation.

*Sample size for index case–based interventions:* We assumed that the NKTA will detect 0.25 case per FC (SD = 0.50) and that the camp will detect 1 case (SD = 1). The latter was based on our previous study in 2010 when a camp search yield 1.5 cases per camp,^8^ assuming the yield would be one-third lower now that the caseload has decreased. The power was set at 80% with 5% level of significance. Using a two-sample mean test, we calculated 18 IC sites per intervention.

*Sample size for entomological assessment:* The sample size for sandfly density measurement was calculated based on the following assumptions: 1) about 55% reduction will be achieved by the intervention; 2) the average sandfly density in the control area after the intervention will be 5.0/household (SD = 5.0, considering over-dispersion in the sandfly data); 3) the average sandfly density in the intervention area after the intervention will be 2.25/household (SD = 2.25); and 4) 80% power, 5% level of significance. Using a two-sample mean test, the required number of households was 32 per intervention for sandfly density measurements. We included 36 households per intervention arm in the study.

### Statistical analysis.

We developed a data management system using EpiInfo version 7 software (CDC, Atlanta, GA) for data entry and data management. Data were checked and cleaned before analysis. Descriptive statistics were obtained for data exploration. Means were compared by parametric and nonparametric methods depending on the distribution of the variables. Proportions were compared by using the Chi-square test.

The yields of newly detected VL, PKDL, and other febrile and skin diseases through the different intervention strategies were expressed as average number of new cases detected per intervention.

To see the efficacy of vector control intervention, the main outcome indicators were sandfly mortality and reduction in indoor sandfly density. Crude intervention effect was then estimated as the difference of the differences, which should be zero if there was no intervention effect and negative if there was a reduction in the intervention groups compared with the control group. The effect of intervention on sandfly count at the household level was calculated as (*B*−*A*)−(*D*−*C*), where *A* = baseline value for the intervention group, *B* = follow-up value for the intervention group, *C* = baseline value for the control group, and *D* = follow-up value for the control group. We calculated the average effect size with 95% confidence interval (CI) at the household level. Furthermore, we used a difference-in-difference regression model to estimate the adjusted intervention effect on female *P. argentipes* reduction after the intervention. As we found that the female *P. argentipes* count fitted a negative binomial distribution, all analyses were carried out under that assumption. A generalized estimating equation model was used to adjust the correlation in data because of longitudinal/repeated measurement in index case–based cluster sampling. In the model, an interaction term of being in the intervention arm at follow-up was included to estimate the effect of the intervention. The regression model has the following structure:

$$\text{Count}=\text{Intercept}+a^{*}\;\text{Treatment}+b^{*}\;\text{Time}+c^{*}\;\text{Interaction}+\text{error,}$$

where treatment is one if it is the intervention and zero if the control; time is one if it is follow-up and zero if baseline; and interaction is one if it is the intervention group at follow-up. The incidence rate ratio (IRR) generated from the exponent of *c*-coefficient and corresponding *P*-values are given in the table. Significance is set at the 5% level and 95% CIs are given. The main outcome variable was count of “female *P. argentipes* sandflies per household” at baseline (2-3 days before intervention) and follow-ups (1, 3, 6, 9, and 12 months after the intervention). The variables that were related to the characteristics of the living room and varied significantly between intervention and control arms were considered as covariates and adjusted in the full model (see Supplemental Table 1).

Bioassay results were reported as percent corrected sandfly mortality, which was calculated using Abbot’s formula^16^ as follows:

$$P=\left[\left(P_{\text{i}}-C\right)\text{/}\left(100-C\right)\right]\times 100\text{,}$$

where *P* = corrected mortality percentage, *P*_i_ = percent observed mortality in insecticide-exposed sandflies, and *C* = percent mortality in control (nonexposed) sandflies.

We performed all the analyses using STATA 10.1 (StataCorp LP, College Station, TX).

### Quality control.

The study activities were monitored by the investigators and by the central level program staff to maintain the unique quality.

### Ethical and environmental considerations.

We obtained approval from the International Centre for Diarrhoeal Disease Research, Bangladesh Ethical Review Committee and from the WHO, Geneva Ethical Review Committee. Informed written consent was obtained from the study participants before conducting interviews and from the household head. Written consent was also obtained from the heads of households for implementing entomological activities in both intervention and control households. Regarding the camp approach, because it is a national program routine activity, written consent was not required.

## Results

### Study profile.

We found 49 VL index cases during the study period from 28 villages in our study area, and deployed 49 index case–based interventions from September 2015 to February 2016, of which 18 were NKTA in 11 villages, 12 were FC + DWL in 10 villages, and 19 were FC + ITN in seven villages (Table 1).

**Table 1:** Study profile and characteristics of index-based interventions

Indicator	NKTA	FC + DWL	FC + ITN
No. of unions	2	2	2
No. of villages	11	10	7
Total number of households (population) in the study villages	12,184 (55,663)	5,570 (24,594)	8,143 (36,869)
No. of index cases (households)	18 (18)	12 (12)	19 (19)
Index case–based vector control activity coverage in % (done/targeted)	–	–	–
Household	93.32 (503/539)	93.05 (308/331)	87.84 (607/691)
Population	93.34 (2,298/2,462)	93.02 (1,360/1,462)	87.82 (2,748/3,129)
Average household (population) per index case–based intervention	30 (139)	28 (118)	36 (168)
Average (SD) family size	4.69 (1.96)	4.27 (1.65)	4.71 (1.93)
Mean (SD) no. of bedrooms/bed-nets*	1.93 (1.11)	1.36 (0.55)	2.16 (1.00)
Mean proportion (in %) of bedrooms/bed-nets* received per intervention per household	90.17 (20.91)	83.60 (24.42)	90.66 (19.21)
Average no. of suspected sandfly breeding places washed with larvicide per index case–based intervention	19.00 (17.34)	N/A	N/A

FC + DWL = fever camp and installation of durable wall lining; FC + ITN = fever camp and insecticide-treated net; NKTA = no kala-azar transmission activity; N/A = not applicable.

* Bed-nets for ITN arms.

The average number of households around an index household which received NKTA ranged from 26 to 28 (Table 1). The coverage of vector control activities was 93% (*n* = 503), 93% (*n* = 308), and 88% (*n* = 607), respectively, for the NKTA, FC + DWL, and FC + ITN arms. The average number of bedrooms per household in the NKTA and FC + DWL arms was 1.9 (SD = 1.11) and 1.4 (SD = 0.55), respectively. About 90% and 84% of these bedrooms received IRS and DWL interventions in the NKTA and FC + DWL arms, respectively. In addition, we found about 19 suspected sandfly breeding spots per index case–based intervention in the NKTA arm, all of which were treated with larvicide to control sandfly larvae. In the FC + ITN arm, the mean number of existing bed-nets per household was 2.2 (SD = 1.0) and 91% of them were impregnated with the K-O Tab 1-2-3 (Table 1).

### Yield of VL, PKDL, and other diseases.

In total, there were 103 camp participants (52 in the FC + DWL arm and 51 in the FC + ITN arm) in 17 camps. The average age of the camp participants was 31.3 years (SD 24.6) and 36.6 years (SD 22.4), and 58% and 63% were females in the FC + DWL and FC + ITN arms, respectively (Table 2). Fifteen (15, 29%) cases in the camp in the FC + DWL arm were referred to the upazila health complex for further management, five of whom were confirmed as PKDL. The FC + ITN arm also referred 19 (37%) of the cases to the upazila health complexes, of whom one was confirmed as VL and two as PKDL. In addition, eight enteric fever and 18 other febrile and skin diseases referred from the camps (10 from FC + DWL and 16 from FC + ITN) were confirmed at the upazila health complex. All confirmed cases were treated at the upazila health complex as per the national guideline. The index case–based house-to-house search in the NKTA arm did not yield any suspected case. In summary, the yield of newly detected VL/PKDL cases per index case–based active case search was 0.0, 0.50, and 0.43, respectively, for the NKTA, FC + DWL, and FC + ITN arms (Table 2).

**Table 2:** Yields of new cases through index-based search in VL-endemic areas in Bangladesh

Indicator	NKTA	FC + DWL	FC + ITN
No. of index case–based searches	18 House-to-house search	10 Camps	7 Camps
No. of attendants to the index case–based house-to-house/camp search	2,505 (539 Households)	64	72
No. of cases with fever for more than 2 weeks or skin lesion like PKDL (suspected cases)	0	52 (camp participants*)	51 (camp participants*)
Mean (SD) age	–	31.65 (24.65)	36.61 (22.37)
Female, % (*n*/*N*)	–	57.70 (30/52)	62.70 (32/51)
Child (< 18 years), % (*n*/*N*)	–	44.20 (23/52)	21.60 (11/51)
Patients referred to the upazila health complex for final diagnosis and treatment, % (*n*/*N*)	–	28.85 (15/52)	37.25 (19/51)
No. of confirmed VL and PDKL cases:	–	5 (VL = 0, PKDL = 5)	3 (VL = 1, PKDL = 2)
Mean (SD) age	–	12.00 (7.71)	26.00 (11.53)
Female, % (*n*/*N*)	–	40.00 (2/5)	66.70 (2/3)
Child (< 18 years), % (*n*/*N*)	–	80.00 (4/5)	33.30 (1/3)
Yield of VL and PDKL cases per intervention	0.0	0.50	0.43
Confirmed cases with other febrile illness and skin disease	0	10 (enteric fever = 3, others† = 7)	16 (enteric fever = 5, others† = 11)

FC + DWL = fever camp and installation of durable wall lining; FC + ITN = fever camp and insecticide-treated net; NKTA = no kala-azar transmission activity; PKDL = post–kala-azar dermal leishmaniasis; VL = visceral leishmaniasis.

* Camp attendant who have chronic fever (fever > 2 weeks) or skin lesion like PKDL.

† Seasonal flu/fungal lesions.

### Efficacy of index case–based sandfly control activities.

The efficacy of the interventions was measured through 1) the reduction of female *P. argentipes* sandfly densities in intervention households compared with the control households, and 2) the sandfly killing ability overtime of treated surfaces by determining the bioavailability of insecticides using bioassay tests.

*Efficacy based on reduction of sandfly density:* At baseline, the average female *P. argentipes* density per household was about 0.67 (SD, 0.93), 0.64 (SD, 1.22), and 0.22 (SD, 0.48) in NKTA, FC + DWL, and FC + INT, respectively. However, the distributions were 0.44 (SD, 0.73), 0.22 (SD, 0.48), and 0.06 (SD, 0.23) in the three different control arms against NKTA, FC + DWL, and FC + ITN intervention arms, respectively. The female *P. argentipes* densities in the intervention and respective control arms were not statistically different at baseline (Table 3). The crude estimated mean (95% CI) reduction of sandfly count per household attributed to IRS plus larvicide and installation of DWL were, respectively, −1.04 (−0.63, −1.42) and −0.56 (−0.33, −0.77) at 12-month follow-up. However, the mean reduction of female *P. argentipes* density in the ITN arm ranged from −6.10 (−3.04, −9.19) to 0.81 (0.01, 1.60) (Table 3).

**Table 3:** Female *Phlebotomus argentipes* sandfly per household and their comparison between intervention vs. control clusters at baseline and follow-up

	Female *P. argentipes* sandfly per household; mean (SD) [*P*-value*]									Effect on count [95% CI]†		
Time	NKTA	Control	*P*-value	FC + DWL	Control	*P*-value	FC + ITN	Control	*P*-value	NKTA	FC + DWL	FC + ITN
Baseline	0.67 (0.93)	0.44 (0.73)	0.340	0.64 (1.22)	0.22 (0.48)	0.272	0.22 (0.48)	0.06 (0.23)	0.074	–	–	–
1-Month follow-up	0.81 (1.12)	2.58 (4.54)	0.037	0.58 (1.02)	1.72 (2.59)	0.009	1.53 (3.59)	7.47 (12.41)	< 0.0001	−2.00 (−0.77, −3.23)	−1.56 (−0.78, −2.33)	−6.10 (−3.04, −9.19)
3-Month follow-up	1.03 (1.13)	5.39 (7.87)	0.001	1.75 (2.61)	2.75 (3.67)	0.097	1.25 (2.71)	5.33 (6.67)	< 0.0001	−4.59 (−2.24, −6.93)	−1.42 (−0.81, −2.02)	−4.24 (−2.83, −5.68)
6-Month follow-up	0.64 (0.83)	1.81 (2.91)	0.199	0.94 (1.71)	1.08 (2.16)	0.993	1.39 (3.03)	0.69 (0.89)	0.682	−1.40 (−0.61, −2.16)	−0.56 (−0.15, −0.95)	0.54 (−0.11, 1.15)
9-Month follow-up	0.69 (1.04)	1.14 (1.46)	0.169	0.78 (1.27)	0.69 (1.26)	0.902	3.03 (4.96)	2.06 (2.35)	0.875	−0.68 (−0.46, −0.87)	−0.33 (−0.09, −0.57)	0.81 (0.01, 1.60)
12-Month follow-up	0.36 (0.83)	1.17 (1.78)	0.013	0.22 (0.72)	0.36 (0.64)	0.147	0.39 (0.84)	0.31 (0.62)	0.785	−1.04 (−0.63, −1.42)	−0.56 (−0.33, −0.77)	−0.08 (−0.06, −0.11)

CI = confidence interval; NKTA = no kala-azar transmission activity; FC + DWL = fever camp and installation of durable wall lining; FC + ITN = fever camp and insecticide-treated net.

* *P*-value for test of mean differences between intervention and control arms.

† Crude estimated effect in female *P. argentipes* sandfly counts attributed by the intervention compared with the control arm. See the calculation in statistical analysis.

**Figure 3.**
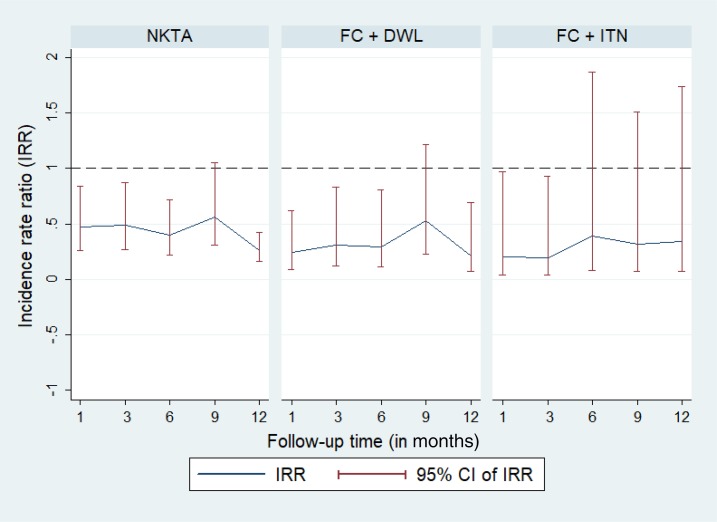
Effect of vector control intervention on female *Phlebotomus argentipes* densities per household. CI = confidence interval; FC + DWL = fever camp and installation of durable wall lining; FC + ITN = fever camp and insecticide-treated net; IRR = incidence rate ratio; NKTA = no kala-azar transmission activity. This figure appears in color at www.ajtmh.org.

We also used the longitudinal regression model to estimate the efficacy of the intervention by adjusting for the effect of covariates and clustering due to the index case–based study design (see Supplemental Table 1 for the list of significant covariates). The adjusted model showed a significant reduction in the incidence rate of female *P. argentipes* sandfly count in the NKTA and FC + DWL arms compared with the control arms up to 12 months post-intervention except at the 9-month follow-up (Table 4, Figure 3). The reduction in the incidence rate of female *P. argentipes* sandfly count was 74% (IRR = 0.26) (*P* < 0.0001) and 79% (IRR = 0.21) (*P* = 0.01) at 12-month follow-up in the NKTA and FC + DWL arms, respectively. The adjusted intervention effect of ITN was statistically significant on reduction of the incidence rate of female *P. argentipes* sandfly count up to 3 months post-intervention but not beyond follow-ups (*P* = 0.242, *P* = 0.151, and *P* = 0.792) (Table 4, Figure 3).

**Table 4:** Effect of intervention on female *Phlebotomus argentipes* densities adjusted for covariates by longitudinal regression analysis

Time/Model	Parameter	IRR [95% CI] (*P*-value)		
		NKTA	FC + DWL	FC + ITN
1-Month follow-up				
Simple model	Crude intervention effect*	0.48 [0.27, 0.85] (0.012)	0.27 [0.11, 0.67] (0.005)	0.19 [0.04, 0.96] 0.044)
Full model	Adjusted intervention effect	0.47 [0.26, 0.84] (0.012)†	0.24 [0.09, 0.62] (0.003)‡	0.20 [0.04, 0.97] (< 0.046)§
3-Month follow-up				
Simple model	Crude intervention effect*	0.46 [0.26, 0.81] (0.007)	0.40 [0.18, 0.91] (0.028)	0.19 [0.04, 0.92] (0.003)
Full model	Adjusted intervention effect	0.49 [0.27, 0.87] (0.016)†	0.31 [0.12, 0.83] (0.020)‡	0.19 [0.04, 0.93] (0.040)§
6-Month follow-up				
Simple model	Crude intervention effect*	0.46 [0.25, 0.87] (0.017)	0.44 [0.19, 0.98] (0.0.47)	0.41 [0.09, 1.93] (0.260)
Full model	Adjusted intervention effect	0.40 [0.22, 0.72 (0.002)†	0.29 [0.11, 0.81] (0.018)‡	0.39 [0.08, 1.87] (0.242)§
9-Month follow-up				
Simple model	Crude intervention effect*	0.59 [0.32, 1.11] (0.103)	0.50 [0.21, 1.14] (0.100)	0.32 [0.07, 1.51] (0.152)
Full model	Adjusted intervention effect	0.56 [0.31, 1.05] (0.073)†	0.53 [0.23, 1.22] (0.139)‡	0.32 [0.07, 1.51] (0.151)§
12-Month follow-up				
Simple model	Crude intervention effect*	0.38 [0.19, 0.77] (0.008)	0.31 [0.11, 0.94] (0.038)	0.34 [0.07, 1.76] (0.201)
Full model	Adjusted intervention effect	0.26 [0.16, 0.42] (< 0.0001)†	0.21 [0.07, 0.69] (0.010)‡	0.34 [0.07, 1.74] (0.792)§

CI = confidence interval; FC + DWL = fever camp and installation of durable wall lining; FC + ITN = fever camp and insecticide-treated net; NKTA = no kala-azar transmission activity; IRR = incidence rate ratio.

* The intervention effect and covariates are tested in two types of longitudinal regression models (generalized estimating equation with negative binomial model) at five different follow-up times: simple not controlling for any covariates and full model controlling covariates. The variables that varied significantly between intervention and control areas are considered as covariates for full model. Only incidence rate ratio with 95% CI and *P*-values for the regression parameter of intervention effect are presented. Regression analysis was performed by considering the clustering effect due to the index-based approach.

† Full model adjusted by the covariates: humidity in the bedroom, mud wall.

‡ Full model adjusted by the covariates: humidity in the bedroom, household head occupation, mud wall.

§ Full model adjusted by the covariates: crack in the wall.

**Figure 4.**
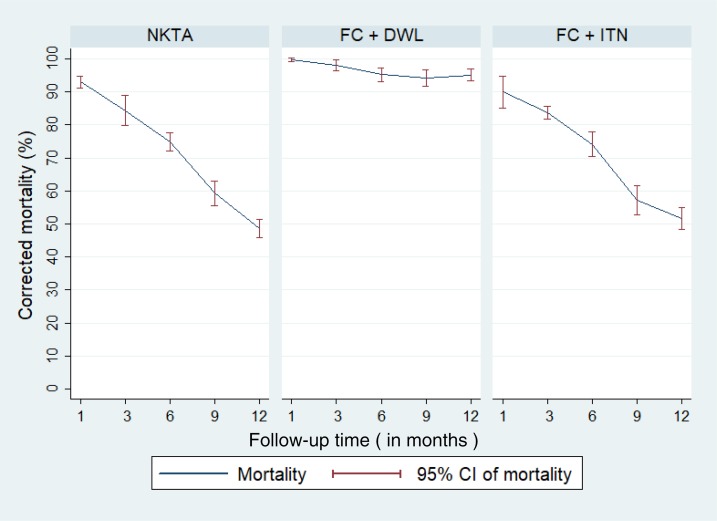
Abbot-corrected *Phlebotomus argentipes* sandfly mortality by the interventions at follow-up periods. CI = confidence interval; FC + DWL = fever camp and installation of durable wall lining; FC + ITN = fever camp and insecticide-treated net; NKTA = no kala-azar transmission activity. This figure appears in color at www.ajtmh.org.

*Efficacy based on WHO cone bioassay:* The corrected mortality of *P. argentipes* sandfly was 95.1% (95% CI: 93.4%, 96.8%), 51.7% (95% CI: 48.3%, 55.1%), and 48.5% (95% CI: 45.8%, 51.3%) on the DWL surface walls, insecticide-treated bed-nets, and IRS surface walls, respectively, at the 12-month follow-up (Figure 4). The killing effect of DWL was consistently high up to 12 months, whereas it decreased over the time for IRS and ITN (Figure 4).

## Discussion

The major finding of our study is that index case–based FCs are more useful for the early detection of VL and PKDL cases than house-to-house search in NKTA in the consolidation phase of the VL elimination program in Bangladesh. The FCs have the additional benefit that they can detect other febrile and skin diseases. Durable wall lining has a stronger and longer lasting killing effect for 12 months or possibly longer than IRS and ITN. Durable wall lining and IRS plus larvicide exert protective efficacy for 12 months and possibly more, whereas ITN (K-O Tab 1-2-3 impregnation) has inconsistent efficacy. The findings of our study are important in informing the Bangladesh national program (NKEP) on the most effective and sustainable approaches for the consolidation and maintenance phases of VL elimination.

Index case–based camps were effective to detect VL and PKDL cases. The yield of new VL and PKDL cases was about 0.5 per camp, which is half the estimated yield based on our previous study conducted in 2011.^10^ This difference is likely because of the sharp fall in VL cases in recent years. Therefore, index case–based FC is a sensitive method to detect cases when they are few and would be missed by the classical NKTA (which did not yield a single case). Of note, the camp approach identified PKDL cases, which are believed to be the Leishmania human reservoir in the interepidemic period. Hence, if deployed systematically, index case–based FC could progressively undermine the transmission potential of VL where it is used. Post–kala-azar dermal leishmaniasis cases are generally difficult to find as the patients are clinically healthy and usually do not seek medical care unless they are stigmatized when skin lesions are obvious and disfiguring. Like general FCs,^9^ index case–based camps bring additional benefits by detecting other febrile and skin diseases, favoring a less vertical and more integrated and sustainable approach in the long run, which could be run in conjunction with the tuberculosis, malaria, and leprosy programs.

This study provides also effective approaches for vector control and transmission reduction. Combining IRS with larviciding in NKTA was found to be effective for 12 months and perhaps longer, which, if confirmed by future studies, is an interesting option, as it lasts at least twice as long as the well-documented 5–6 months with IRS alone.^17,18^ Previous studies reported that larvicides are effective in reducing pupal productivity of larval habitats and reducing indoor and outdoor resting vectors.^19,20^ Moreover, a cluster-randomized controlled trial in Bangladesh showed that the combination of several vector control tools such IRS with alpha-cypermethrin, outdoor spraying with chlorpyrifos, and commercially made long-lasting insecticide-treated bed-net (LLIN) and bed-net impregnation with a slow-release insecticide tablet (K-O Tab 1-2-3) can extend efficacy to up to 2 years. Combination of LLIN and outdoor spraying with chlorpyrifos was most effective in reducing VL vector densities for 22 months or longer.^21^

DWL is another promising tool for vector control which was found to be highly effective in controlling sandfly in the Indian subcontinent.^14,22^ In this study, index case–based DWL intervention was also found to be highly effective in reducing sandfly densities at the household level. Whether it will be possible to apply DWL systematically will depend on its cost, as it is at present higher than that of other sandfly control tools.^14,23^ A cost-effectiveness analysis of the DWL approach that also considers its prolonged efficacy is required. We have yet unpublished observations that DWL efficacy persists for at least 2 years.

In the present study, ITN was not as effective as in a previous study^11^ in reducing sandfly densities. This might be because of the scale of implementation of this intervention: whereas the earlier study used mass impregnation of the bed-nets across the whole community, in the current study, the approach was limited to 60 houses around the index case.

Our study is not without its limitations. We had fewer index cases than planned, which meant that only two of the three arms (NKTA and FC + ITN) had the minimum calculated 18 index case–based interventions, whereas the FC + DWL arm had only 12 index cases, which might have affected the estimation of the yield of new cases using this approach. We could not have an epidemiological end-point because of the currently low incidence of VL in Bangladesh; therefore, we could not measure the effects of reduced vector densities on parasite transmission and new infections and cases. While not yet available, we are working to estimate the cost-effectiveness of these intervention packages, based on available cost data.^10,14,22^

In conclusion, the Bangladesh NKEP should consider index case–based FCs for the early detection of VL and PKDL cases during the consolidation and maintenance phases of the elimination program, and either DWL or IRS plus larvicide for sandfly control.

## Supplementary Material

Supplemental figure

## References

[1] Alvar J Velez ID Bern C Herrero M Desjeux P Cano J Jannin J den Boer M; WHO Leishmaniasis Control Team, 2012 Leishmaniasis worldwide and global estimates of its incidence. PloS One 7: e35671. 22693548 10.1371/journal.pone.0035671 PMC3365071

[2] World Health Organization, 2005 Regional Technical Advisory Group on Kala-Azar Elimination. Report of the First Meeting, Manesar, Haaryana, 20–23 December 2004. New Delhi, India: Regional Office for South-East Asia.

[3] Ahmed B-N Nabi SG Rahman M Selim S Bashar A Rashid MM Lira FY Choudhury TA Mondal D, 2014 Kala-azar (visceral leishmaniasis) elimination in Bangladesh: successes and challenges. Curr Trop Med Rep 1: 163–169.

[4] Bern C Chowdhury R, 2006 The epidemiology of visceral leishmaniasis in Bangladesh: prospects for improved control. Indian J Med Res 123: 275–288. 16778310

[5] Rahman R Bangali M Kabir H Naher F Mahboob S, 2008 *Kala-azar Situation in Bangladesh*, pp. 11–12. National guideline and training module for Kala-azar elimination in Bangladesh. Directorate General of Health Services, Ministry of Health and Family Welfare, Dhaka, Bangladesh.

[6] Molina R Ghosh D Carrillo E Monnerat S Bern C Mondal D Alvar J, 2017 Infectivity of post-kala-azar dermal leishmaniasis patients to sand flies: revisiting a proof of concept in the context of the kala-azar elimination program in the Indian subcontinent. Clin Infect Dis 65: 150–153. 28520851 10.1093/cid/cix245 PMC5848257

[7] NKEP, CDC, 2016 National Guideline for Kala-azar Case Management in Bangladesh. Dhaka, Bangladesh: Directorate General of Health Services.

[8] Singh SP 2011 Options for active case detection of visceral leishmaniasis in endemic districts of India, Nepal and Bangladesh, comparing yield, feasibility and costs. PLoS Negl Trop Dis 5: e960. 21347452 10.1371/journal.pntd.0000960 PMC3035672

[9] Banjara MR Kroeger A Huda MM Kumar V Gurung CK Das ML Rijal S Das P Mondal D, 2015 Feasibility of a combined camp approach for vector control together with active case detection of visceral leishmaniasis, post kala-azar dermal leishmaniasis, tuberculosis, leprosy and malaria in Bangladesh, India and Nepal: an exploratory study. Trans R Soc Trop Med Hyg 109: 408–415. 25918216 10.1093/trstmh/trv031 PMC4499944

[10] Huda MM 2012 Active case detection in national visceral leishmaniasis elimination programs in Bangladesh, India, and Nepal: feasibility, performance and costs. BMC Public Health 12: 1001. 23164318 10.1186/1471-2458-12-1001 PMC3533526

[11] Mondal D 2010 Insecticide-treated bed nets in rural Bangladesh: their potential role in the visceral leishmaniasis elimination programme. Trop Med Int Health 15: 1382–1389. 20946233 10.1111/j.1365-3156.2010.02635.x

[12] Mondal D Huda MM Karmoker MK Ghosh D Matlashewski G Nabi SG Kroeger A, 2013 Reducing visceral leishmaniasis by insecticide impregnation of bed-nets, Bangladesh. Emerg Infect Dis 19: 1131–1134. 23764246 10.3201/eid1907.120932 PMC3713966

[13] Messenger LA 2012 Multicentre studies of insecticide-treated durable wall lining in Africa and South-East Asia: entomological efficacy and household acceptability during one year of field use. Malar J 11: 358. 23107112 10.1186/1475-2875-11-358 PMC3547731

[14] Mondal D 2016 Efficacy, safety and cost of insecticide treated wall lining, insecticide treated bed nets and indoor wall wash with lime for visceral leishmaniasis vector control in the Indian sub-continent: a multi-country cluster randomized controlled trial. PLoS Negl Trop Dis 10: e0004932. 27533097 10.1371/journal.pntd.0004932 PMC4988640

[15] World Health Organization, UNICEF, 2010 *Monitoring and evaluation tool kit for indoor residual spraying: Kala-azar elimination in Bangladesh, India and Nepal* Available at: http://www.who.int/tdr/publications/documents/irs_toolkit.pdf.

[16] Abbott WS, 1987 A method of computing the effectiveness of an insecticide. 1925. J Am Mosq Control Assoc 3: 302–303. 3333059

[17] World Health Organization Expert Committee, 1990 *Control of the Leishmaniases*. World Health Organ, Tech Rep Ser, 793. 2124015

[18] Najera JA Zaim M, 2001 *Malaria Vector Control: Insecticides for Indoor Residual Spraying*. World Health Organization document WHO/CDS/WHOPES/2001.3 Geneva, Switzerland: World Health Organization.

[19] Afrane YA Mweresa NG Wanjala CL Gilbreath Iii TM Zhou G Lee MC Githeko AK Yan G, 2016 Evaluation of long-lasting microbial larvicide for malaria vector control in Kenya. Malar J 15: 577. 27903292 10.1186/s12936-016-1626-6 PMC5131428

[20] Fillinger U Knols BG Becker N, 2003 Efficacy and efficiency of new *Bacillus thuringiensis* var israelensis and *Bacillus sphaericus* formulations against Afrotropical anophelines in Western Kenya. Trop Med Int Health 8: 37–47. 12535249 10.1046/j.1365-3156.2003.00979.x

[21] Chowdhury R 2017 Control of *Phlebotomus argentipes* (Diptera: Psychodidae) sand fly in Bangladesh: a cluster randomized controlled trial. PLoS Negl Trop Dis 11: e0005890. 28873425 10.1371/journal.pntd.0005890 PMC5600390

[22] Huda MM 2016 Entomological efficacy of durable wall lining with reduced wall surface coverage for strengthening visceral leishmaniasis vector control in Bangladesh, India and Nepal. BMC Infect Dis 16: 539. 27716091 10.1186/s12879-016-1881-8 PMC5052807

[23] Das M Banjara M Chowdhury R Kumar V Rijal S Joshi A Akhter S Das P Kroeger A, 2008 Visceral leishmaniasis on the Indian sub-continent: a multi-centre study of the costs of three interventions for the control of the sandfly vector, *Phlebotomus argentipes*. Ann Trop Med Parasitol 102: 729–741. 19000390 10.1179/136485908X355274

