# Supplementary material for: Intervention Packages for Early Visceral Leishmaniasis Case Detection and Sandfly Control in Bangladesh: A Comparative Analysis
Source: Am J Trop Med Hyg. 2018 Nov 19;100(1):97–107. doi: 10.4269/ajtmh.18-0290 (PMC6335927; doi:10.4269/ajtmh.18-0290)
Supplement: Supplementary file 1 [file tpmd180290.SD1.pdf]

Supplemental Table 1. Baseline characteristics of the household where entomological efficacy were measured

	Female *P. argentipes* sandfly per household; Mean (SD) [P-value*]								
	NKTA Arm			FC+DWL Arm			FC+ITN Arm		
**Indicator**	**Intervention**	**Control**	***P*-value**	**Intervention**	**Control**	***P*-value**	**Intervention**	**Control**	***P*-value**
Unskilled household head; % (n)	30.56 (11)	19.44 (7)	0.276	41.67 (15)	13.89 (5)	0.009	30.56 (11)	25.00 (9)	0.599
Housing materials:									
Mud wall	8.33 (3)	25.00 (9)	0.058	11.11 (4)	30.56 (11)	0.042	13.89 (5)	2.78 (1)	0.088
Mud floor	72.22 (26)	86.11 (31)	0.147	100.0 (36)	88.89 (32)	0.040	80.56 (29)	86.11 (31)	0.527
Damp floor	0.0 (0)	0.0 (0)	–	2.78 (1)	0.0 (0)	1.000	2.78 (1)	0.0 (0)	1.000
Cracks in wall	11.11 (4)	25.0 (9)	0.126	25.0 (9)	33.33 (12)	0.437	25.0 (9)	5.56 (2)	0.022
Mean (SD) humidity in the living room	75.68 (5.97)	70.43 (3.72)	< 0.001	79.18 (5.29)	69.14 (4.75)	< 0.001	68.38 (5.80)	68.11 (2.98)	0.809
Having cattle shed	25.0 (9)	44.44 (16)	0.083	50.0 (18)	61.11 (22)	0.343	47.22 (17)	47.22 (17)	1.000

NKTA = No Kala-azar Transmission Activity; FC+DWL = Fever Camp and Installation of Durable Wall Lining; FC+ITN = Fever Camp and Insecticide Treated Net.
